# The longitudinal trajectory of CSF sTREM2: the alzheimer’s disease neuroimaging initiative

**DOI:** 10.1186/s13195-024-01506-8

**Published:** 2024-06-26

**Authors:** Yu Wang, Meijie Ye, Qianqian Ji, Qi Liu, Xiaowei Xu, Yiqiang Zhan

**Affiliations:** 1 Department of Epidemiology, School of Public Health (Shenzhen), Sun Yat-Sen University, Shenzhen, China; 2 Department of Neurology, The Seventh Affiliated Hospital of Sun Yat-Sen University, Shenzhen, China; 3 Institute of Environmental Medicine, Karolinska Institutet, Stockholm, Sweden

**Keywords:** CSF sTREM2, Trajectory, Sex, Longitudinal study

## Abstract

**Background:**

The soluble triggering receptor expressed on myeloid cells 2 (sTREM2) in cerebrospinal fluid (CSF) is considered a biomarker of microglia activity. The objective of this study was to investigate the trajectory of CSF sTREM2 levels over time and examine its association with sex.

**Methods:**

A total of 1,017 participants from the Alzheimer’s Disease Neuroimaging Initiative Study (ADNI) with at least one CSF sTREM2 record were included. The trajectory of CSF sTREM2 was analyzed using a growth curve model. The association between CSF sTREM2 levels and sex was assessed using linear mixed-effect models.

**Results:**

CSF sTREM2 levels were increased with age over time (*P* < 0.0001). No significant sex difference was observed in sTREM2 levels across the entire sample; however, among the *APOE* ε4 allele carriers, women exhibited significantly higher sTREM2 levels than men (β = 0.146, *P* = 0.002).

**Conclusion:**

Our findings highlight the association between CSF sTREM2 levels and age-related increments, underscoring the potential influence of aging on sTREM2 dynamics. Furthermore, our observations indicate a noteworthy association between sex and CSF sTREM2 levels, particularly in individuals carrying the *APOE* ε4 allele.

**Supplementary Information:**

The online version contains supplementary material available at 10.1186/s13195-024-01506-8.

## Introduction

The triggering receptor expressed on myeloid cell 2 (TREM2) is exclusively expressed by microglia in the brain [1]. As a transmembrane receptor, TREM2 mediates protective functions on microglia, serving as a regulator of phagocytosis and suppression of inflammatory reactivity [2, 3]. Further, *TREM2* mutations are known to be a risk factor for sporadic Alzheimer’s disease (AD) [4]. TREM2 extracellular domain is capable of binding various ligands related to microglia, such as lipopolysaccharides (LPS), phospholipids, high-density lipoprotein cholesterol (HDL-C), low-density lipoprotein cholesterol (LDL-C), ApoE, apoptotic neurons, and soluble amyloid-beta (Aβ) [5]. A soluble form of TREM2 (sTREM2) is generated through cleaving the TREM2 extracellular domain by ADAMs (a disintegrin and metalloproteinase) after ligand binding [6]. Although cerebrospinal fluid (CSF) sTREM2 is considered a biomarker of microglia activity [7, 8], the biological role of sTREM2 in AD is poorly understood [9–11]. Previous studies suggested that sTREM2 could exert neuroprotective effects by enhancing the clearance effect of microglia for Aβ plaque in earlier stages of dementia [12, 13]. However, it is unclear whether microglia can efficiently regulate Aβ levels, because microglia in a morphologically dystrophic state with loss of surveillance function have been found in late-stage AD, leading to reduced Aβ clearance in late-onset AD patients [14].

Age, sex, and *APOE* ε4 status are major risk factors for dementia, and previous studies examined their relationships with CSF sTREM2 levels [15–18]. These studies suggested aging was associated with sTREM2, the sex differences were inconclusive. While *APOE ε*4 could change the structure of TREM2 and influence TREM2 mRNA expression, its association with sTREM2 levels was not significant [19, 20]. Notably, elevations in sTREM2 levels have been observed in AD, but the highest sTREM2 levels were found during the early phase of AD [15, 18, 21]. A few studies utilizing the ATN profiles have depicted sTREM2 in contrast to amyloid and tau aggregating [9]. These potential changes of sTREM2 over cognitive impairment might be attributed to that microglia function and density exhibit considerable population and spatial heterogeneity in the brain [22, 23]. However, few previous studies have specifically investigated the trajectory of CSF sTREM2 levels over time [17]. Studying the longitudinal dynamic trajectories of sTREM2 can have significant implications for understanding the biological processes underlying neurodegenerative diseases, particularly those involving microglial activation and neuroinflammation [24]. Further, it has the potential to deepen our understanding of AD, uncover novel therapeutic targets, and pave the way for precision medicine approaches tailored to individual trajectories [25–27]. Thus, it is necessary to clearly describe the trajectory of sTREM2 over time to understand better the biological implications of CSF sTREM2 levels.

To this end, we aimed to investigate the trajectories of CSF sTREM2 and to examine the association of CSF sTREM2 with sex using a large longitudinal cohort.

## Methods

### Study population

Participants were selected from the Alzheimer’s Disease Neuroimaging Initiative (ADNI) study, which consisted of 1,031 participants who had CSF sTREM2 measured. The ADNI cohort is a multi-site longitudinal study that began in 2004 to validate biomarkers in dementia or AD clinical trials. A total of 4,139 participants were recruited with follow-up assessments performed every six months in the US and Canada between 2004 and 2021. During the follow-up visits, demographic information, cognitive assessments, and biomarker records were obtained within a six-month time window. More detail about the ADNI study design and the participant recruitment procedure has been described elsewhere [28]. The individuals from ADNI cohort with at least one CSF sTREM2 record were included. Figure S1 presents the selection process for the analytical sample and data used from the ADNI cohort. The final study sample for the present study comprised 1,017 participants, including 305 CN individuals, 519 with MCI, and 193 with dementia at baseline. Participants in MCI or dementia subgroups were older and more likely to be men. At the baseline, the CSF sTREM2 had a median value of 3,598 (interquartile range: 2,490, 5,174) pg/ml. During the follow-up period, 404 participants had sTREM2 measured twice and over. Detailed characteristics of the study participants are described in Table S1.

### Measurement of CSF sTREM2 and other biomarkers

The levels of CSF sTREM2 were measured by a sandwich-based ELISA approach using the mesoscale discovery (MSD) platform, which had been elaborated and validated previously [29]. For the measurements of CSF sTREM2, a subset of the samples from the same participants were measured separately in the Piccio group and Haass group. The ADNI biomarker core flagged 25 individuals, who were outside of the 98% prediction tolerance levels that was constructed by predicting the linear regression model between the two measurements. According to their suggestion, we removed these 25 individuals in subsequent analyses, and *MSD sTREM2corrected* was utilized for the following statistical analyses [30].

The levels of β-amyloid (1–42) (Aβ_1−42_), total tau (t-Tau), and phosphotau (181P) (p-Tau) in CSF by the ADNI Biomarker Core were measured using the Elecsys β-amyloid (1–42) (Roche Diagnostics) CSF, the Elecsys Total-Tau CSF, and the Elecsys phosphotau (181P) CSF immunoassays, respectively [31].

### Classification of cognitive status

Clinical cognitive status was classified by ADNI investigators as follows: normal cognitive (CN: Mini-Mental State Examination [MMSE] ≥ 24, Clinical Dementia Rating [CDR] = 0, and non-depressed), mild cognitive impairment (MCI: MMSE ≥ 24, CDR = 0.5, objective memory-impairment on education-adjusted Wechsler Memory Scale II, and preserved activities of daily living) or dementia (MMSE = 20–26, CDR ≥ 0.5, and NINCDS/ADRDA criteria for probable AD). Following the 2018 NIA-AA criteria, each ADNI participant was assigned into groups by the ATN framework. In the present study, the ATN biomarker profiles include three biomarkers: “A” as Aβ aggregation, “T” as tauopathy, and “N” as neurodegeneration. Aβ-positive (A+) participants were those with CSF Aβ_1−42_ levels < 976.6 pg/ml. Tau-positive (T+) participants referred to those with p-tau > 21.8 pg/ml. Neurodegenerative-positive (N+) participants were those with t-tau > 245 pg/ml [32]. Given CSF p-Tau_181P_ and CSF t-Tau are highly correlated, the “T” (tau pathology) and “N” (neurodegeneration) groups were merged [30]. The TN- was defined as both the normal range of aggregated tau and neurodegeneration. The TN + presented those with tau pathology or neurodegeneration.

### Covariates

The covariates included in the analyses were selected based on various factors previously reported to be associated with CSF sTREM2, inflammation in the brain, as well as imbalanced demographic characteristics comparing the recruited study sample to the ADNI entire sample. These included age, sex, race/ethnicity, *TREM2* rare variant carrying status, *APOE* ε4 allele, educational attainment, smoking, marital status, clinical cognitive status, and AD core biomarkers. Race/ethnicity was classified as non-Hispanic white and others (non-Hispanic black, non-Hispanic others, and Hispanic). According to *APOE* ε4 allele status, participants were classified as *APOE* ε4 non-carrier *(APOE* ε2/ε2 or *APOE* ε2/ε3 or *APOE* ε3/ε2 or *APOE* ε3/ε3*)*, *APOE* ε4 heterozygote (*APOE* ε3/ε4 or *APOE* ε2/ε4), *APOE* ε4 homozygote (*APOE* ε4/ε4*)*. Participants were stratified into non-smokers if they reported no history of cigarette smoking and as smokers if they reported a history of cigarette smoking. Marital status was classified as either married or others (widowed, divorced, never married, and unknown).

### Statistical analysis

The distributions of demographic characteristics and biomarkers levels at baseline among three cognitive status groups (CN, MCI, and dementia) were compared by Pearson chi-square test for categorical variables, one-way analyses of covariance (ANCOVA) for continuous variables, and Mann-Whitney U test for ordinal categorical variables. To allow for interpretation on a relative scale and to account for non-normal distribution, levels of CSF sTREM2, CSF Aβ_1−42_, CSF t-Tau, and CSF p-Tau underwent log_e_ transformation.

Linear mixed-effect models were employed to fit growth curves to the repeated measurements of CSF sTREM2. In these models, each trajectory parameter (intercept and slope) was allowed to vary from individual to individual (as random effects) and to vary by the trajectory, baseline demography, and cognitive impairment factors for each participant (as fixed effects). To give the coefficients a meaningful interpretation at zero, and to avoid multicollinearity, variables were centered in the models. Covariates were added in a stepwise fashion into a series of models. Model 1 included age as fixed effects with an additional random slope and intercept for age. Model 2 added sex as covariate. Model 3 further added race/ethnicity, *TREM2* rare variant carrying status, *APOE* ε4 carrying status (fixed effects). Model 4 additionally adjusted educational attainment, smoking status, marital status, and clinical cognitive status. Model 5 and model 6 additionally controlled for CSF Aβ_1−42_, CSF t-Tau, CSF p-Tau, and the p-Tau/Aβ_1−42_ ratio. The goodness of fit of the model was evaluated with Akaike’s information criteria (AIC). Finally, interaction analyses and ANCOVA analyses were conducted to test for longitudinal interaction effects between age, sex, and each covariate to determine whether CSF sTREM2 trajectories varied according to specific demographic and other factors. The primary codes for the model implementation have been provided in the Supplementary files.

All statistical analyses were performed using R 4.2.2. All tests were 2-tailed, with a significance level of α = 0.05.

## Results

### Distribution of CSF sTREM2 at baseline

**Fig. 1:**
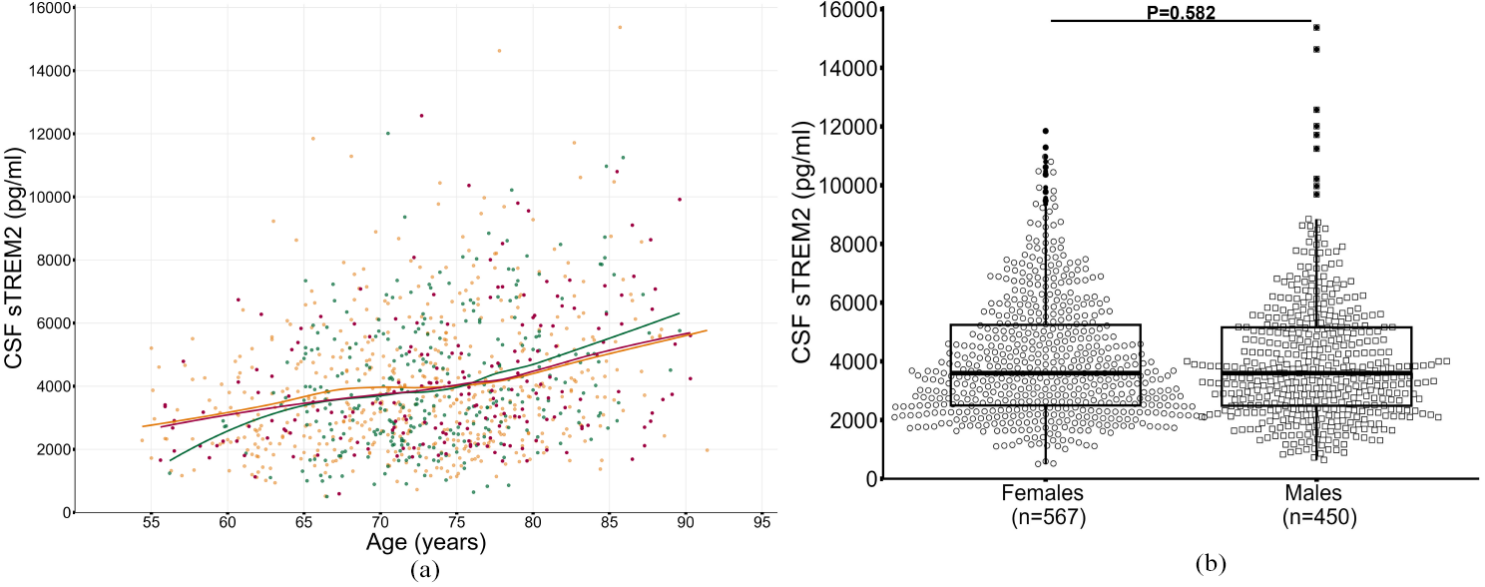
(**A**) The dynamic change of CSF sTREM2 over age among CN, MCI, and dementia groups at baseline. Solid lines represent the regression lines for the three clinical cognitive performance subgroups: the green line and points denote the cognitively normal group, the orange line and points denote the MCI group, and the red line and points denote the dementia group. (**B**) The distribution of CSF sTREM2 levels by sex at baseline. *Abbreviations* CN, cognitively normal controls; MCI, mild cognitive impairment

Figure 1A illustrates that baseline levels of CSF sTREM2 were significantly associated with age (β = 77.26, *P* < 0.001, Table 1). Specifically, the yearly increment in CSF sTREM2 was 82 pg/ml for those aged 55–64 years and 33 pg/ml for those aged 65–74 years. CSF sTREM2 dramatically increased for individuals over 75 years old (*P*_interaction_=0.023), with an average annual rise of 133 pg/ml. In addition, mean CSF sTREM2 concentrations were significantly higher in A-T + group (A-T+, 5302 pg/ml > A + T+, 4284 pg/ml > A-T-, 3555 pg/ml > A + T-, 2809 pg/ml, Figure S2A) and non-Hispanic whites (4129 pg/ml vs. 3174 pg/ml, *P* < 0.001, Figure S2B) than others, whereas CSF sTREM2 levels did not vary substantially by sex, clinical cognitive status, or *APOE* ε4 carrier status (Fig. 1B, Figure S2C, Figure S2D).

### The longitudinal trajectory of CSF sTREM2 over time

**Fig. 2:**
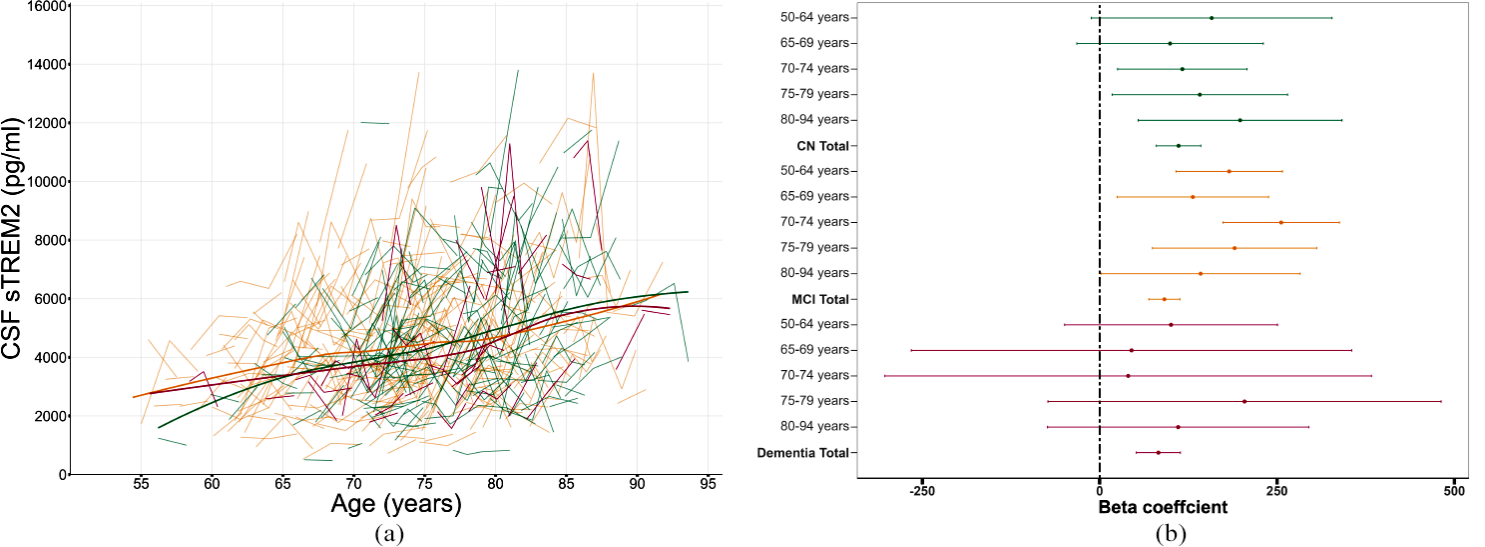
(**A**) Longitudinal trajectories of CSF sTREM2 over time among CN, MCI, and dementia groups. Thin solid lines connecting points indicate changes in CSF sTREM2 of follow-up for each individual. Thick solid lines represent the trajectories of CSF sTREM2 using cubic spline models for three clinical cognitive performance subgroups at baseline: the green lines signify the cognitively normal group, the orange lines signify the MCI group, and the red lines signify the dementia group. (**B**) The correlation coefficients between age and CSF sTREM2 among different age ranges. The regression coefficients and confidence intervals were obtained using linear mixed-effect models, without adjusting for covariates. *Abbreviations* CN, cognitively normal controls; MCI, mild cognitive impairment; CI, confidence interval

The longitudinal trajectories of CSF sTREM2 levels using cubic spline models by clinical cognitive status were displayed in Fig. 2A. The increasing trends of these trajectories exhibited slight variations among CN, MCI, and dementia groups. Notably, above the age of 75, we did not detect a significantly faster growth rate in longitudinal sTREM2 levels (*P*_interaction_=0.389, Table 1; Fig. 2B). Furthermore, the observed slight differences in sTREM2 trajectories among patients with MCI or dementia were not statistically significant compared with cognitively normal individuals (β=-0.005, *P =* 0.299; β=-0.006, *P =* 0.305, Table S2).

**Table 1:** Annual change rates of CSF sTREM2 by baseline age interval and consecutive slope difference using baseline and longitudinal data, ADNI cohort, United States and Canada, 2004–2021

Baseline age interval	Size/ observations	Mean^a^	Slope^b^	Se^c^	*P* ^d^	Slope difference (β (*P* value))^e^	Mean^a^	Slope^b^	Se^c^	*P* ^d^	Slope difference (β (*P* value))^e^
		**Baseline data (CN)** ^**f**^					**Longitudinal data (CN)** ^**g**^				
50–64	11/19	2678.68	162.54	143.08	0.285	-	2566.85	157.89	86.55	0.090	-
65–74	173/294	3722.57	30.27	49.43	0.541	-132.27 (0.560)	3935.93	90.11	31.24	0.004	-75.73 (0.581)
≥ 75	121/247	4674.34	161.75	57.00	0.005	131.48 (0.079)	4994.55	151.48	39.07	< 0.001	60.65 (0.223)
		**Baseline data (MCI)** ^**f**^					**Longitudinal data (MCI)** ^**g**^				
50–64	93/165	3161.63	61.99	60.06	0.305	-	3356.53	182.46	38.30	< 0.001	-
65–74	228/433	3965.49	22.03	45.73	0.630	-39.96 (0.633)	4235.47	151.70	28.81	< 0.001	-32.21 (0.531)
≥ 75	198/345	4420.29	126.01	46.71	0.008	103.98 (0.116)	4812.92	150.34	35.79	< 0.001	-2.91 (0.949)
		**Baseline data (dementia)** ^**f**^					**Longitudinal data (dementia)** ^**g**^				
50–64	34/37	3230.83	127.94	81.79	0.128	-	3213.05	100.58	76.55	0.198	-
65–74	56/77	3783.69	101.74	105.23	0.338	-26.21 (0.854)	3752.38	68.61	85.41	0.424	-33.42 (0.784)
≥ 75	103/135	4619.19	125.95	51.42	0.016	24.22 (0.842)	4723.13	137.52	49.18	0.006	52.80 (0.611)
		**Baseline data (entire sample)** ^**f**^					**Longitudinal data (entire sample)** ^**g**^				
50–64	138/221	3140.18	82.42	46.14	0.076	-	3268.37	167.04	32.26	< 0.001	-
65–74	457/804	3851.25	33.02	31.84	0.300	-49.40 (0.443)	4079.45	125.93	20.57	< 0.001	-44.10 (0.293)
≥ 75	422/727	4541.68	133.38	29.43	< 0.001	100.36 (0.023)	4859.65	145.90	23.29	< 0.001	27.12 (0.389)
Total	1072/1752	4041.26	77.26	8.68	< 0.001		4362.55	90.81	7.91	< 0.001	

^a^Mean: the mean value of CSF sTREM2 levels within the baseline age interval, pg/ml

^b^Slope: the annual rate of change in sTREM2 levels within the baseline age interval, pg/(ml·year)

^c^Se: the standard error of the annual rate of change in sTREM2 levels within the baseline age interval, pg/(ml·year)

^d^*P*: the *P* value of the annual rate of change in sTREM2 levels within the baseline age interval

^e^Slope difference (β (P value)): the difference in annual rates of change in sTREM2 levels between the older age interval and the prior younger age interval, pg/(ml·year^2^)

^f^All multivariate linear regression models had no adjustment for covariates

^g^All linear mixed-effect models included age as fixed effects with an additional random slope and intercept for age

*Abbreviations* CN, cognitively normal; MCI, mild cognitive impairment; CSF, cerebrospinal fluid; sTREM2, soluble TREM2; β, regression coefficient; Se, standard deviation

### The longitudinal association of CSF sTREM2 with age

**Fig. 3:**
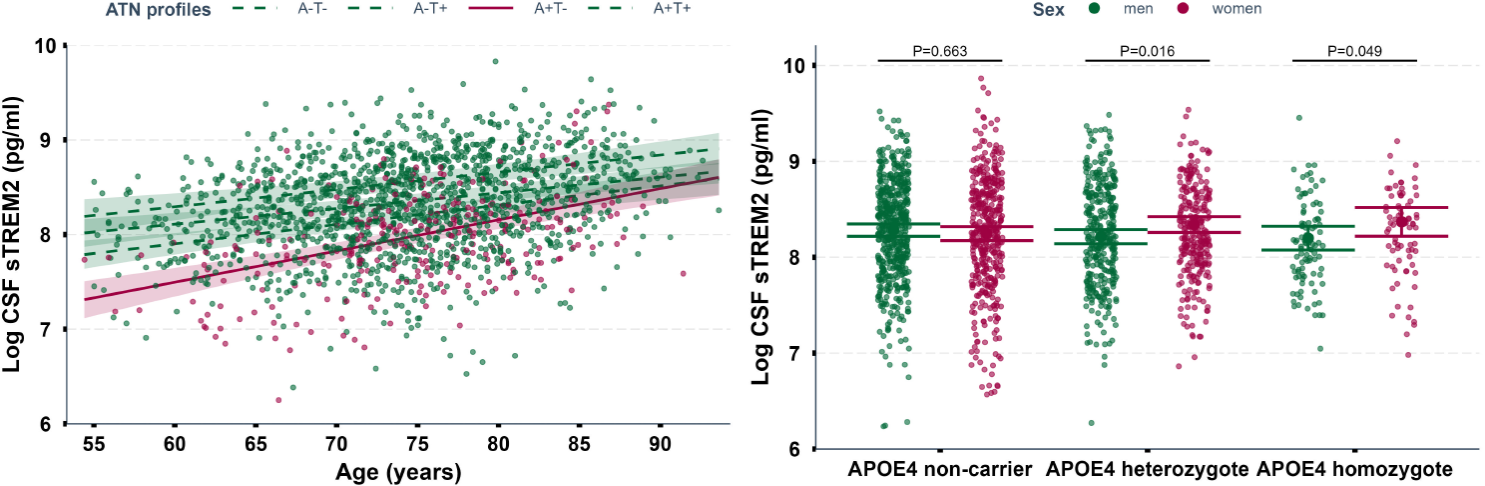
(**A**) Longitudinal interaction between the ATN profiles and age in CSF sTREM2 levels. This figure illustrates the interaction effect of ATN profiles on longitudinal age-related changes in CSF sTREM2 within a mixed-effect model considering covariates, using the ‘interact_plot’ function from the ‘interaction’ package. Red points represent the A + T- group, and green points represent the other groups (A-T-, A-T+, A + T+). Regression lines are shown for the A + T- group (red, solid) and the other groups (green, dashed), with standard errors represented by red and green shading. (**B**) Longitudinal interaction between *APOE* ε4 and sex in CSF sTREM2 levels. This figure illustrates the interaction effect of *APOE* ε4 on longitudinal sex-related changes in CSF sTREM2 within a mixed-effect model considering covariates, using the ‘cat_plot’ function from the ‘interaction’ package. Red points represent the female group, and green points represent the male group

The overall results of fixed-effect models showed a strong positive association between age and CSF sTREM2 (*P* < 0.0001, Table 2). The average annual change of sTREM2 was 2.2% (the average rate increase was 90 pg/ml per year), controlling for sex, race/ethnicity, *TREM2* rare variant status, *APOE* ε4 allele status, educational attainment, smoking, marital status, and clinical cognitive status in model 4 (Table 2). This finding remained consistent after controlling for CSF Aβ_1−42_, CSF t-Tau, CSF p-Tau, and p-Tau/Aβ_1−42_ ratio (model 5, model 6). Moreover, A + T- group exhibited faster age-related increase in sTREM2 levels than others (Table 3; Fig. 3A).

**Table 2:** Longitudinal trajectory of CSF sTREM2 and the association of CSF sTREM2 with sex using longitudinal data, ADNI cohort, United States and Canada, 2004–2021

Variables	Model 1^a^	Model 2^b^	Model 3^c^	Model 4^d^	Model 5^e^	Model 6^f^
Age (years)	0.023 (0.002) ^***g^	0.023 (0.002) ^***^	0.022 (0.002) ^***^	0.022 (0.002) ^***^	0.018 (0.002) ^***^	0.020 (0.002) ^***^
Sex (referent: men)		0.013 (0.031)	0.025 (0.031)	0.043 (0.034)	-0.037 (0.029)	0.016 (0.031)
Race/ethnicity (referent: Non-Hispanic white)			-0.336 (0.053) ^***^	-0.329 (0.053) ^***^	-0.220 (0.046) ^***^	-0.304 (0.053) ^***^
*TREM2* rare variant carrying status			-0.107 (0.095)	-0.112 (0.096)	-0.130 (0.081)	-0.150 (0.094)
*APOE* ε4 allele status^h^						
*APOE* ε4 non-carrier			Ref	Ref	Ref	Ref
*APOE* ε4 heterozygote			0.007 (0.033)	0.000 (0.034)	-0.036 (0.031)	-0.048 (0.034)
*APOE* ε4 homozygote			-0.001 (0.052)	-0.012 (0.054)	-0.043 (0.052)	-0.127 (0.057) ^*^
Educational attainment				0.005 (0.006)	0.008 (0.005)	
Smoking				-0.012 (0.031)	-0.021 (0.027)	
Marital status (referent: married)				-0.031 (0.039)	-0.029 (0.033)	
Clinical cognitive status						
CN				Ref	Ref	
MCI				-0.017 (0.029)	-0.044 (0.026)	
Dementia				0.036 (0.035)	-0.029 (0.034)	
CSF Aβ_1−42_					0.205 (0.029) ^***^	
CSF t-Tau					0.474 (0.137) ^***^	
CSF p-Tau					0.083 (0.124)	
The p-Tau/Aβ_1−42_ ratio						2.268 (0.481)^***^
Size	1017	1017	1012	1005	997	1012
Observations	1752	1752	1742	1731	1691	1742
AIC	1747	1754	1730	1745	1406	1682

^a^Model 1 included age as fixed effects with an additional random slope and intercept for age

^b^Model 2 added sex as covariate

^c^Model 3 further added race/ethnicity (referent: non-Hispanic white), *TREM2* rare variant carrying status (any *TREM2* rare variant vs. no *TREM2* rare variant), *APOE* ε4 carrying status (one E4 vs. no E4, two E4 vs. no E4) (fixed effects)

^d^Model 4 additionally adjusted educational attainment (continuous), smoking status (ever smoker vs. never smoker), marital status (referent: married), clinical cognitive status (referent: CN)

^e^Model 5 additionally controlled for CSF Aβ1–42, CSF t-Tau, CSF p-Tau

^f^Model 6 further introduced the p-Tau/Aβ1–42 ratio based on model 3

^g^Values are shown as regression coefficient (standard error)

^h^*APOE* ε4 allele status: *APOE* ε4 non-carrier, *APOE* ε2/ε2 or *APOE* ε2/ε3 or *APOE* ε3/ε2 or *APOE* ε3/ε3; *APOE* ε4 heterozygote, *APOE* ε3/ε4 or *APOE* ε2/ε4; *APOE* ε4 homozygote, *APOE* ε4/ε4

^*^represeted statistically significant. ^*^Significant at *P* < 0.05. ^**^Significant at *P* < 0.01. ^***^Significant at *P* < 0.001

*Abbreviations* CN, cognitively normal; MCI, mild cognitive impairment; CSF, cerebrospinal fluid; Aβ1–42, amyloid-β1–42; t-Tau, total tau; sTREM2, soluble TREM2

### The longitudinal association of CSF sTREM2 with sex

Compared with men, women did not have significantly higher levels of CSF sTREM2 controlling for age (β = 0.013, *P* = 0.683, Table 2), and the difference remained nonsignificant after further adjusting for race/ethnicity, *TREM2* rare variant status, *APOE* ε4 allele status, educational attainment, smoking, marital status, and clinical cognitive status (β = 0.043, *P* = 0.203). Nevertheless, the interaction effects between *APOE* ε4 and sex, as well as between educational attainment and sex, were pronounced in model 4 (β = 0.159, *P* = 0.016; β = 0.207, *P* = 0.049, Table 3; Fig. 3B). In subgroup analysis stratified by *APOE* ε4 carrying status, a significant increase in sTREM2 levels was observed in women compared to men among *APOE* ε4 carriers (β = 0.146, *P* = 0.002, Table S3). In an exploratory stratified analysis by sex, females carrying the *APOE* ε4 allele, as well as those with higher educational attainment, exhibited significantly higher sTREM2 levels after covariate adjustment (β = 0.104, *P* = 0.036; β = 0.021, *P* = 0.019, Table S3).

**Table 3:** Interaction effect analysis of age and covariates, sex and covariates in CSF sTREM2 using longitudinal data, ADNI cohort, United States and Canada, 2004–2021

Interaction term	Age × covariates^a^		Sex × covariates^a^	
	β (95% CI)	*P* value	β (95% CI)	*P* value
Sex (Women)	-0.006 (-0.061, 0.049)	0.830	-	-
Clinical cognitive status				
CN	Ref		Ref	
MCI	-0.009 (-0.060, 0.042)	0.725	0.066 (-0.044, 0.176)	0.241
Dementia	-0.012 (-0.077, 0.053)	0.722	0.025 (-0.106, 0.156)	0.711
*AOPE ε4* allele status^b^				
*AOPE* ε4 non-carrier	Ref		Ref	
*AOPE* ε4 heterozygote	-0.039 (-0.098, 0.020)	0.201	0.159 (0.030, 0.288)	0.016
*AOPE* ε4 homozygote	-0.057 (-0.155, 0.041)	0.252	0.207 (0.001, 0.413)	0.049
The ATN classification^c^				
A + T-	Ref		Ref	
A-T-	-0.087 (-0.171, -0.003)	0.043	0.017 (-0.169, 0.203)	0.860
A-T+	-0.105 (-0.191, -0.019)	0.016	0.128 (-0.068, 0.324)	0.203
A + T+	-0.121 (-0.197, -0.045)	0.002	0.107 (-0.065, 0.279)	0.223
Educational attainment (years)	-0.008 (-0.018, 0.002)	0.106	0.024 (0.00, 0.048)	0.041
CSF Aβ_1−42_	0.014 (-0.031, 0.059)	0.545	0.044 (-0.056, 0.144)	0.387
CSF t-Tau	-0.024 (-0.079, 0.031)	0.394	-0.024 (-0.079, 0.031)	0.394
CSF p-Tau	-0.024 (-0.073, 0.025)	0.338	0.034 (-0.074, 0.142)	0.538
The p-Tau/Aβ_1−42_ ratio	-0.719 (-1.521, 0.083)	0.079	-0.808 (-2.462, 0.846)	0.339

^a^Models adjusted for age, sex, race/ethnicity, education levels, *TREM2* rare variant carrying status, *APOE* ε4 allele status, smoking status, marital status, and clinical cognitive status as fixed effects with an additional random slope and intercept for age

^b^*APOE* ε4 allele status: *AOPE* ε4 non-carrier, *APOE* ε2/ε2 or *APOE* ε2/ε3 or *APOE* ε3/ε2 or *APOE* ε3/ε3; *AOPE* ε4 heterozygote, *APOE* ε3/ε4 or *APOE* ε2/ε4; *AOPE* ε4 homozygote, *APOE* ε4/ε4

^c^The ATN classification: The ATN classification system included 3 biomarker subgroups: “A” as Aβ aggregation, “T” as tauopathy, and “N” as neurodegeneration. Aβ-positive (A+) participants were those with CSF Aβ_1−42_ levels < 976.6 pg/ml. Tau-positive (T+) participants referred to those who had a p-Tau > 21.8 pg/ml. Neurodegenerative-positive (N+) individuals were those with t-Tau > 245 pg/ml

*Abbreviations* CN, cognitively normal; MCI, mild cognitive impairment; CSF, cerebrospinal fluid; Aβ_1−42_, amyloid-β1–42; t-Tau, total tau; p-Tau, phosphorylated tau_181 − p_; sTREM2, soluble TREM2; β, regression coefficient; CI, confidence interval

## Discussion

In the present study, our prospective analysis of 1,017 individuals from the ADNI cohort demonstrated the longitudinal trajectories of CSF sTREM2 levels. CSF sTREM2 levels were increased over age, and strong positive linear correlations were seen among CN, MCI, and dementia groups. While the trajectories of sTREM2 did not show significant variation across the three clinical cognitive groups, there was a notably faster rate of increase in sTREM2 levels following the onset of amyloid pathology in the early stages of AD. Furthermore, women exhibited significantly higher sTREM2 levels compared to men among the *APOE* ε4 allele carriers, but this sex difference was not significant across the entire sample.

A few cross-sectional studies have reported a strong positive association between age and CSF sTREM2 [16–18, 33–35]. Moreover, this association has also been observed in several cohorts of Parkinson’s disease [36, 37]. Further, in a hospital-based longitudinal study of Swedish and Norwegian participants, Henjum et al. found that CSF sTREM2 levels were related to age only in cognitively normal participants, not in those with MCI or AD [17]. In contrast, elevated levels of sTREM2 with age were reported for CN, MCI, and dementia participants in a pooled study with five memory clinics, which are consistent with our results [15]. Around 75% of sTREM2 is generated by the shedding of full-length TREM2 [38]. An age-related increase in TREM2 mRNA expression was demonstrated in previous transcriptome analyses in both elderly AD mouse models [39] and human AD brains [19, 40, 41]. Therefore, CSF sTREM2 elevated with aging would mainly be attributed to TREM2 expression.

Regarding the sTREM2 trajectory, in contrast to the results of Suárez-Calvet et al., our study did not observe a faster increase in sTREM2 levels among individuals with MCI or dementia compared to cognitively normal individuals [42]. This divergence in findings might be attributed to the different age ranges of the study samples (40–100 years vs. 54–94 years). In ATN profiles, although the individuals with Aβ pathology without tau pathology or neurodegeneration (A + T-) at baseline exhibited lower sTREM2 levels compared to other stages, a more rapid increase rate in sTREM2 was observed in those with this initial Aβ pathology stage during follow-up. This is consistent with findings from the Dominantly Inherited Alzheimer Network (DIAN) study, in which Morenas-Rodríguez et al. reported that a high baseline amyloid burden was a predictor of the annual increase rates of sTREM2 in carriers of pathogenic variants (*APP*/*PSEN1*/*PSEN2*). These observations indicated there might be an accelerated increase in sTREM2 in the early stages of AD [43]. One possible explanation is that the inflammatory response to Aβ deposition within the brain might trigger higher amounts of tau to be released into CSF, thereby further influencing microglia reactivity processes and subsequent neurodegeneration [21, 44].

In our dataset, the trajectories of sTREM2 demonstrated a notable acceleration after the age of 75, particularly within the cognitively normal subgroup. This pattern of change is consistent with observations of age-related proteins in plasma, which have shown similar acceleration at critical ages (34, 60, and 78) in the aging process, implicating blood pathways and bone morphogenetic protein signaling [45]. The observed increase in CSF sTREM2 levels after the age of 75 may be attributed to factors such as race, ethnicity, or varying levels of tau dynamic change in the CSF [46]. Our study sample exhibited a higher proportion of non-Hispanic whites in the 80–94 age group, followed by the 75–79 and 70–74 age groups. Additionally, a slight acceleration in the growth rate of t-Tau was observed after the age of 75 (with increases of 5.78, 6.14, and 6.32 pg/ml per year in the age groups of 70–74, 75–79, and 80–94, respectively). Furthermore, it has been documented that inflammatory stimuli predominantly increase TREM2 expression in vivo [47]. Various basal pro-inflammatory cytokines exhibit nonlinear trajectories, with specific acceleration inflection points, such as IL-6 and IL-10 in plasma at approximately the age of 70 [48–50], and YKL-40 in CSF around 60 years old [51]. These findings collectively suggest that inflammation and microglial dysfunction may accelerate in older individuals.

A previous study reported a significantly higher sTREM2 in men (*n* = 106) compared to women (*n* = 112) in DIAN [52], similar to another study based on Alzheimer’s Disease Research Center (ADRC) cohort (*P* = 0.017) [18]. However, a recent European study found that CSF sTREM2 levels were not associated with sex (*P* = 0.179) [42]. Results from the Chinese Alzheimer’s Biomarker and Lifestyle (CABLE) study also found that sTREM2 concentration was not affected by sex (*P* = 0.576) [16]. In Parkinson’s disease, inconsistent results were found regarding CSF sTREM2 and sex. While Parkinson’s Progression Markers Initiative data reported no association (*P* = 0.888) [37], the Pacific Udall Center Cohort revealed significantly higher sTREM2 levels in men than in women (*P* < 0.0001) [36]. The inconsistent findings from different cohorts were partly due to the different sample sizes examined. A possible explanation would be that there is a differential neuroinflammatory response to p-Tau accumulation between men and women [53, 54], and that late-life changes in estrogen levels among women have a direct effect on tau [55, 56]. Studies with larger samples are needed to further examine this association.

Exploratory stratified analysis by sex showed that women carrying the *APOE* ε4 allele had higher CSF sTREM2 levels, but this pattern was not observed in men. These results were consistent with previous studies demonstrating an interaction between *APOE* ε4 allele and sex [57, 58]. Wood et al. discovered that the *APOE* ε2 allele selectively protected men against cognitive decline compared to the *APOE* ε3/ε3 but was not as protective in women [59]. In a previous examination of the ADNI cohort, a significant disparity was observed in brain hypometabolism and cortical thinning among female *APOE* ε4 carriers, who exhibited more profound effects compared to female non-carriers [60]. Several studies have consistently reported an *APOE* ε4-dependent sex difference in the deposition of p-Tau and t-Tau [61–64]. Wang et al. further expanded on this finding, revealing that *APOE* ε4 carriers in females displayed significantly higher tau burden in specific brain regions, including the hippocampus, entorhinal cortex, and parahippocampal cortex, compared to *APOE* ε4 carriers in males. Notably, the authors also identified a positive correlation between microglial activity and the escalation of amyloid load, particularly among female *APOE* ε4 carriers [65]. This association further underscores the complexity of the *APOE* ε4-sex interaction in the pathogenesis of AD. Underlying mechanisms could be that estrogen following menopause promoted the release of APOE from microglia [66], enhancing the *APOE* ε4 effect on Aβ [67], thereby further stimulating microglia activity [65]. Another possibility is that the interaction between *APOE* ε4 genotype and sex might directly affect the microglial activity in the brain and could accelerate the risk of AD [68]. In addition, our results also showed that the association between educational attainment and CSF sTREM2 was only significant in females. This is in line with previous research which demonstrated that cognitive reserve may protect against amyloid-related cognitive impairment [69–71]. The sex differences in neuroprotective effects of cognitive reserve may be related to CSF sTREM2 [72, 73].

Our analysis did not yield significant differences in sTREM2 levels between the cognitively normal group and the Alzheimer’s disease or mild cognitive impairment groups. This observation could be biological plausibility, given the varying severity of cognitive impairment across different cohorts. In our study, the majority of AD cases exhibited very mild MMSE scores (23 ± 2) [21] and it is noteworthy that MMSE scores in AD patients have been correlated with levels of microglial activation [74]. Furthermore, the levels of aberrant microglial activation are influenced not only by clinical cognitive status or neuroinflammation [75], but also directly affected by the status of Aβ and tau [76]. Within the ATN profiles of our study population, 89% of AD patients exhibited abnormal Aβ pathology, while 32% and 40% of the CN group displayed abnormal Aβ or tau pathology, respectively. It is worth noting that during the follow-up period, the dementia group demonstrated higher sTREM2 levels than the cognitively normal group, although this difference did not reach a significant level after adjusting for age. This finding is consistent with previous analyses [15, 26], underscoring the potential significance of age as a key factor in understanding the function and status of sTREM2.

Our study has several strengths that address the methodological shortcomings of earlier studies. An important strength of this study was the relatively large population-based cohort with repeated measurements of sTREM2 and its comprehensive record of the demographic characteristics, assessment of dementia, and detailed measurement of CSF sTREM2, Aβ_1−42_, and t-Tau level. The rich information on the participants allowed us to consider more comprehensively covariates and obtain a more thorough understanding of various aspects associated with CSF sTREM2. Furthermore, the record involved the measurement date of sTREM2 and date of birth, which enabled us to employ longitudinal data and mixed-effect models. The mixed-effect model had the advantage of being able to add more covariates that may have collinearity but are essential since most predictive variables had been centered before being added to models. Importantly, given the substantial inter-individual variability in sTREM2 levels [77, 78], the longitudinal study design provides an opportunity to accurately explore the relationships between sTREM2 and age, sex, as well as to compare differences in trajectories of CSF sTREM2 across different clinical cognitive status.

Our study also has limitations. ADNI cohort did not provide inclusion and exclusion criteria for the participants that measured CSF sTREM2. Thus, it is difficult to interpret the distribution of clinical cognitive status in the study sample. For example, there were more MCI participants than CN participants at baseline. Therefore, we cannot rule out the possibility that selection bias influenced our results. In the current study, conclusions pertaining to microglial activity based on sTREM2 measurements must be approached with caution. The sTREM2 assay employed in the ADNI cohort quantified total sTREM2 levels in the cerebrospinal fluid, which comprises both directly transcribed sTREM2 from sTREM2 mRNA and sTREM2 derived from TREM2 shedding [43]. Notably, approximately 25% of the total sTREM2 quantified originates directly from transcription, rather than via TREM2 shedding, thereby reducing the specificity of the measured sTREM2 as a sole indicator of microglial activity [38]. Moreover, sTREM2 itself possesses distinct functional roles, such as preventing the aggregation of Aβ and promoting microglial phagocytosis of Aβ [13, 79]. While interpreting the results, it is important to acknowledge the limitation of skewed ethnic representation within our cohort. The considerable discrepancy between the Non-white Hispanic subgroup and other ethnicities, raises potential concerns regarding the generalizability of our findings. This disparity underscores the need for caution when extrapolating the study outcomes to populations with diverse ethnic backgrounds [46, 80, 81]. Finally, although mixed-effect models were used to analyze this longitudinal data, the number of follow-up visits was small.

## Conclusions

In conclusion, our findings highlight the association between CSF sTREM2 levels and age-related increments, underscoring the potential influence of aging on sTREM2 dynamics. Furthermore, our observations indicate a noteworthy association between sex and CSF sTREM2 levels, particularly in individuals carrying the *APOE* ε4 allele. These findings emphasize the importance of considering sex-specific differences in sTREM2 levels, especially in the context of genetic risk factors for neurodegenerative diseases. Further research into the interplay between sex, genetic factors, and sTREM2 dynamics is warranted to elucidate the underlying mechanisms and potential implications for disease progression and therapeutic interventions.

### Electronic supplementary material

Below is the link to the electronic supplementary material.

Supplementary Material 1

## Data Availability

No datasets were generated or analysed during the current study.

## Abbreviations

sTREM2: Soluble triggering receptor expressed on myeloid cells 2
CSF: Cerebrospinal fluid; ADNI: the Alzheimer’s Disease Neuroimaging Initiative Study
AD: Alzheimer’s disease
LPS: Lipopolysaccharides
HDL-C: High-density lipoprotein cholesterol
ApoE: Apolipoprotein E
LDL-C: Low-density lipoprotein cholesterol
Aβ_1−42_: β-amyloid (1–42)
t-Tau: Total tau
p-Tau: Phosphotau (181P)
MMSE: Mini-Mental State Examination
CDR: Clinical Dementia Rating
NINCDS: National Institute of Neurological and Communicative Disorders
ADRDA: Stroke and the Alzheimer’s Disease and Related Disorders Association
CN: Normal cognitive
MCI: Mild cognitive impairment
ANCOVA: One-way analyses of covariance
AIC: The Akaike information criterion
DIAN: Dominantly Inherited Alzheimer Network
ADRC: Alzheimer’s Disease Research Center
CABLE: The Chinese Alzheimer’s Biomarker and Lifestyle
