# Supplementary material for: The longitudinal trajectory of CSF sTREM2: the alzheimer’s disease neuroimaging initiative
Source: Alzheimers Res Ther. 2024 Jun 26;16:138. doi: 10.1186/s13195-024-01506-8 (PMC11202383; doi:10.1186/s13195-024-01506-8)
Supplement: Supplementary file 1 — Supplementary Material 1 [file 13195_2024_1506_MOESM1_ESM.docx]

| **Table S1.** Demographic Characteristics of Participants at Baseline, ADNI Cohort, United States and Canada, 2004-2021. | | | | | |
| --- | --- | --- | --- | --- | --- |
| Characteristics | Total  (n=1017) | CN  (n=305) | MCI  (n=519) | Dementia  (n=193) | *P* Value |
| Mean Age (years)^a^ | 73.1±7.30 | 73.6±5.95 | 72.3±7.48 | 74.5±8.39 | <0.001 |
| Sex (Women)^b^ | 567 (55.8%) | 151 (49.5%) | 305 (58.8%) | 111 (57.5%) | 0.031 |
| Race/ethnicity (Non-Hispanic white)^c^ | 919 (90.4%) | 268 (87.9%) | 475 (91.5%) | 176 (91.2%) | 0.231 |
| Educational attainment (years) | 16.0 (2.8) | 16.4 (2.7) | 16.0 (2.8) | 15.5 (2.8) | 0.005 |
| *TREM2* rare variant carrying status | 24 (2.4%) | 6 (2.0%) | 14 (2.7%) | 4 (2.1%) | 0.823 |
| *APOE* ε4 allele status^d^ |  |  |  |  | <0.001 |
| *APOE* ε4 non-carrier | 538 (52.9%) | 223 (73.1%) | 253 (48.7%) | 62 (32.1%) |  |
| *APOE* ε4 heterozygote | 373 (36.7%) | 76 (24.9%) | 208 (40.1%) | 89 (46.1%) |  |
| *APOE* ε4 homozygote | 106 (10.4%) | 6 (1.97%) | 58 (11.2%) | 42 (21.8%) |  |
| Smokers | 403 (39.7%) | 124 (40.7%) | 201 (38.7%) | 78 (40.6%) | 0.823 |
| Marital status (married) | 785 (77.6%) | 213 (69.8%) | 407 (79.3%) | 165 (85.5%) | <0.001 |
| CSF Aβ_1-42_ (pg/ml)^e^ | 860 (603-1405) | 1321 (870-1721) | 841 (610-1328) | 594 (464-750) | <0.001 |
| CSF t-Tau (pg/ml) | 258 (196-345) | 215 (175-281) | 257 (196-341) | 331 (267-445) | <0.001 |
| CSF p-Tau (pg/ml) | 24.0 (17.5-33.6) | 19.6 (15.7-26.1) | 23.9 (17.7-34.0) | 33.2 (25.3-45.1) | <0.001 |
| CSF sTREM2 (pg/ml) | 3598 (2490-5174) | 3632 (2604-5225) | 3574 (2413-5109) | 3585 (2610-5281) | 0.5803 |
| Mean person-visits | 2.33 | 2.51 | 2.39 | 1.68 | <0.001 |
| The ANT classification^f^ |  |  |  |  | <0.001 |
| A+T- | 250 (24.6%) | 127 (41.6%) | 117 (22.5%) | 6 (3.1%) |  |
| A-T- | 181 (17.8%) | 77 (25.2%) | 90 (17.3%) | 14 (7.3%) |  |
| A-T+ | 165 (16.2%) | 54 (17.7%) | 91 (17.5%) | 20 (10.4%) |  |
| A+T+ | 411 (40.4%) | 44 (14.4%) | 217 (41.8%) | 150 (77.7%) |  |
| Change in cognitive status | 284 (28%) | 74 (24%) | 210 (40%) | ~~-~~ |  |
| ^a^Continuous variables with normal distribution were expressed as means ± standard deviation.  ^b^Categorical variables were expressed as counts (percentages).  ^c^Race/ethnicity was dichotomized into non-Hispanic white and other categories (non-Hispanic black, non-Hispanic others, and Hispanic).  ^d^*APOE ε4* allele status: *APOE ε4* non-carrier, *APOE ε2/ε2* or *APOE ε2/ε3* or *APOE ε3/ε2* or *APOE ε3/ε3*; *APOE* *ε4* allele carrier, *APOE ε3/ε4* or *APOE ε2/ε4* or *APOE ε4/ε4*.  ^e^Continuous variables with non-normal distributions were expressed as median (interquartile range).  ^f^The ANT classification: The ATN classification system included 3 biomarker subgroups: “A” as Aβ aggregation, “T” as tauopathy, and “N” as neurodegeneration. Aβ-positive (A+) participants were those with CSF Aβ_1-42_ levels < 976.6 pg/ml. Tau-positive (T+) participants referred to those who had a p-Tau > 21.8 pg/ml. Neurodegenerative-positive (N+) individuals were those with t-Tau > 245 pg/ml.  Abbreviations: CN, cognitively normal; MCI, mild cognitive impairment; CSF, cerebrospinal fluid; Aβ_1-42_, amyloid-β1-42; t-Tau, total tau; p-Tau, phosphorylated tau_181-p_; sTREM2, soluble TREM2. | | | | | |

| **Table S2.** Longitudinal Trajectory of CSF sTREM2 among Clinical Cognitive Status at Baseline Using Longitudinal Data, ADNI Cohort, United States and Canada, 2004-2021. | | | | | | |
| --- | --- | --- | --- | --- | --- | --- |
|  | Model 1^a^ | | Model 2^b^ | | Model 3^c^ | |
|  | β (se) | *P* value | β (se) | *P* value | β (se) | *P* value |
| Clinical cognitive status at baseline |  |  |  |  |  |  |
| CN | Ref |  | Ref |  | Ref |  |
| MCI | 0.007 (0.037) | 0.855 | 0.039 (0.036) | 0.271 | 0.038 (0.036) | 0.282 |
| Dementia | 0.012 (0.048) | 0.797 | 0.005 (0.046) | 0.916 | 0.006 (0.046) | 0.893 |
| Age |  |  | 0.023 (0.002) | <0.001 | 0.027 (0.004) | <0.001 |
| Age × Clinical cognitive status at baseline |  |  |  |  |  |  |
| Age × CN |  |  |  |  | Ref |  |
| Age × MCI |  |  |  |  | -0.005 (0.005) | 0.299 |
| Age × Dementia |  |  |  |  | -0.006 (0.006) | 0.305 |
| ^a^Model 1 included clinical cognitive status at baseline(referent: CN).  ^b^Model 2 further added age as fixed effects with an additional random slope and intercept for age.  ^c^Model 3 additionally introduced the interaction effect of age×clinical cognitive status at baseline.  Abbreviations: CN, cognitively normal; MCI, mild cognitive impairment; CSF, cerebrospinal fluid; β, regression coefficient; se, standard deviation. | | | | | | |

| **Table S3.** Exploratory Stratified Analysis for CSF sTREM2 by *APOE ε4* Allele Status and Sex Using Longitudinal Data, ADNI Cohort, United States and Canada, 2004-2021. | | | | | | | | |
| --- | --- | --- | --- | --- | --- | --- | --- | --- |
|  | *APOE* ε4 allele status^a^ | | | | Sex^b^ | | | |
|  | *APOE* ε4 non-carrier | | *APOE* ε4 carrier | | Men^a^ | | Women^b^ | |
|  | β (se) | *P* value | β (se) | *P* value | β (se) | *P* value | β (se) | *P* value |
| Age (years) | 0.175(0.020) | <0.001 | 0.149(0.021) | <0.001 | 0.158(0.019) | <0.001 | 0.175(0.022) | <0.001 |
| Sex (referent: men) | -0.056(0.048) | 0.243 | 0.146(0.047) | 0.002 |  |  |  |  |
| Race/ethnicity (referent: Non-Hispanic white) | -0.390 (0.070) | <0.001 | -0.249(0.083) | 0.003 | -0.393(0.076) | <0.001 | -0.297(0.076) | <0.001 |
| *TREM2* rare variant carrying status | 0.020 (0.160) | 0.900 | -0.174(0.116) | 0.136 | -0.300(0.137) | 0.029 | 0.046(0.134) | 0.731 |
| *APOE* ε4 allele status (referent: *APOE* ε4 non-carrier) |  |  |  |  | -0.079(0.041) | 0.059 | 0.104(0.050) | 0.036 |
| Educational attainment | -0.005(0.008) | 0.523 | 0.018(0.008) | 0.027 | -0.006(0.008) | 0.413 | 0.021(0.009) | 0.019 |
| Smoking | -0.050(0.044) | 0.258 | 0.034(0.044) | 0.443 | -0.017(0.041) | 0.678 | -0.032(0.050) | 0.526 |
| Marital status (referent: married) | -0.028(0.053) | 0.605 | -0.014(0.056) | 0.798 | -0.095(0.062) | 0.129 | 0.007(0.051) | 0.894 |
| Clinical cognitive status |  |  |  |  |  |  |  |  |
| CN |  |  |  |  |  |  |  |  |
| MCI | -0.050(0.037) | 0.183 | 0.034(0.044) | 0.442 | -0.043(0.038) | 0.251 | 0.007(0.044) | 0.872 |
| Dementia | -0.001(0.051) | 0.980 | 0.093(0.050) | 0.062 | 0.032(0.046) | 0.485 | 0.028(0.053) | 0.592 |
| ^a^The linear mixed-effect models adjusted for age, sex, race/ethnicity, education levels, *TREM2* rare variant carrying status, smoking status, marital status, and clinical cognitive status (fixed effects) plus random slope (age) and intercept.  ^b^The linear mixed-effect models adjusted for age, race/ethnicity, education levels, *TREM2* rare variant carrying status, *APOE ε4* allele status, smoking status, marital status, and clinical cognitive status (fixed effects) plus random slope (age) and intercept.  Abbreviations: CN, cognitively normal; MCI, mild cognitive impairment; APOE, Apolipoprotein E; CSF, cerebrospinal fluid; Aβ_1-42_, amyloid-β1–42; t-Tau, total tau; p-Tau, phosphorylated tau_181-p_; sTREM2, soluble TREM2; β, regression coefficient; se, standard deviation. | | | | | | | | |

**
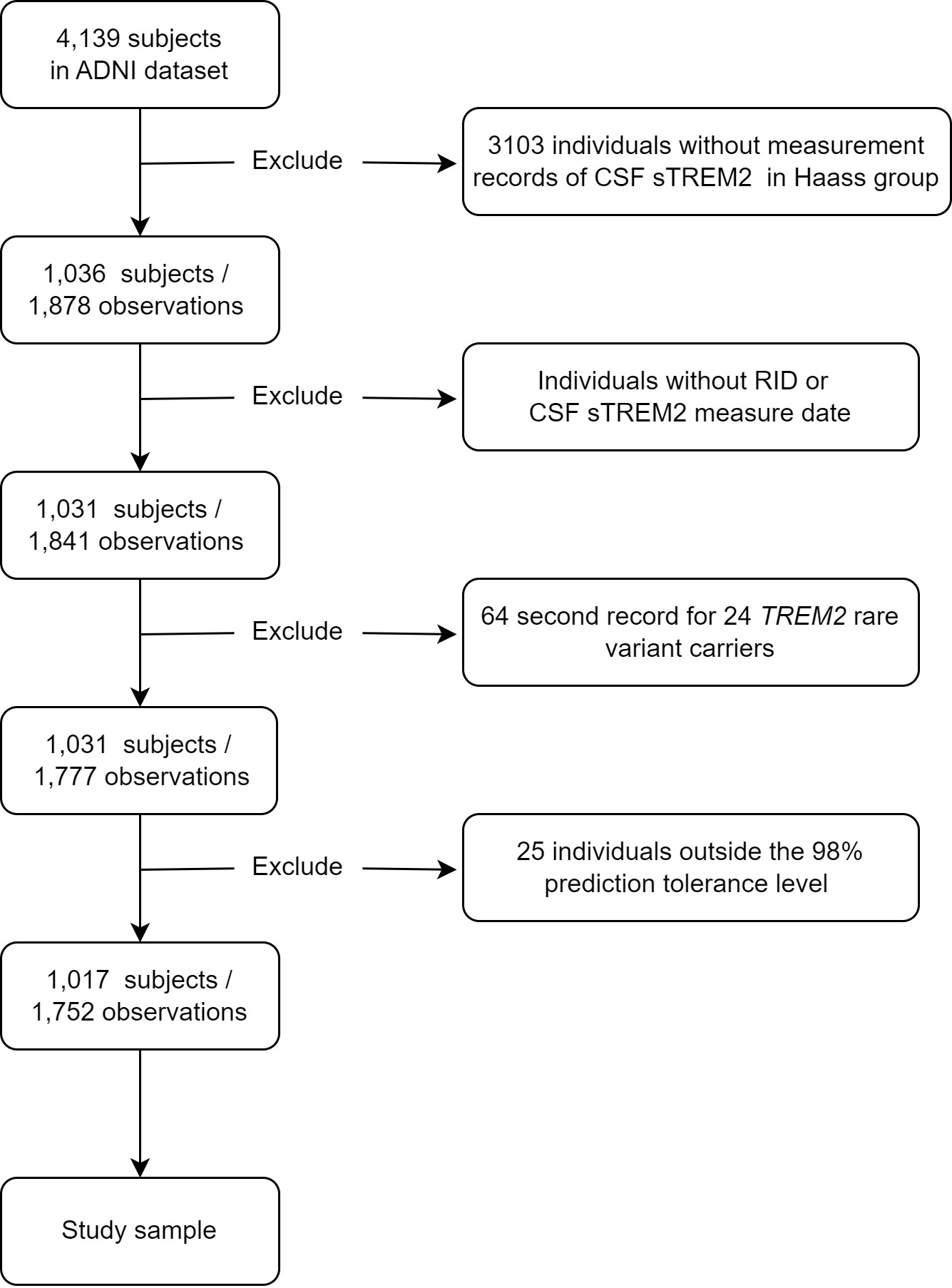
**

**Figure S1.** Selection of the final analytical sample from the Alzheimer's Disease Neuroimaging Initiative Study.


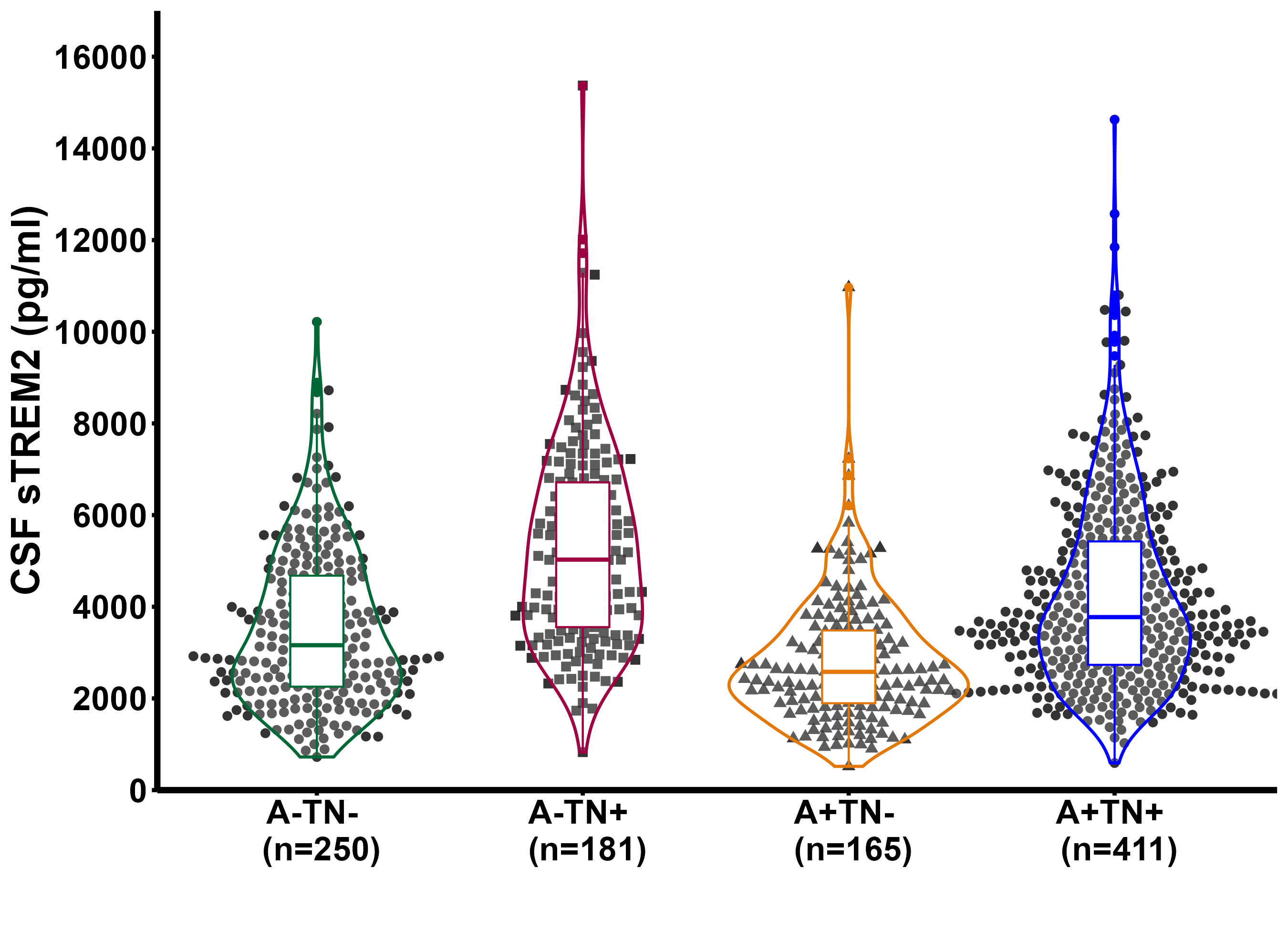

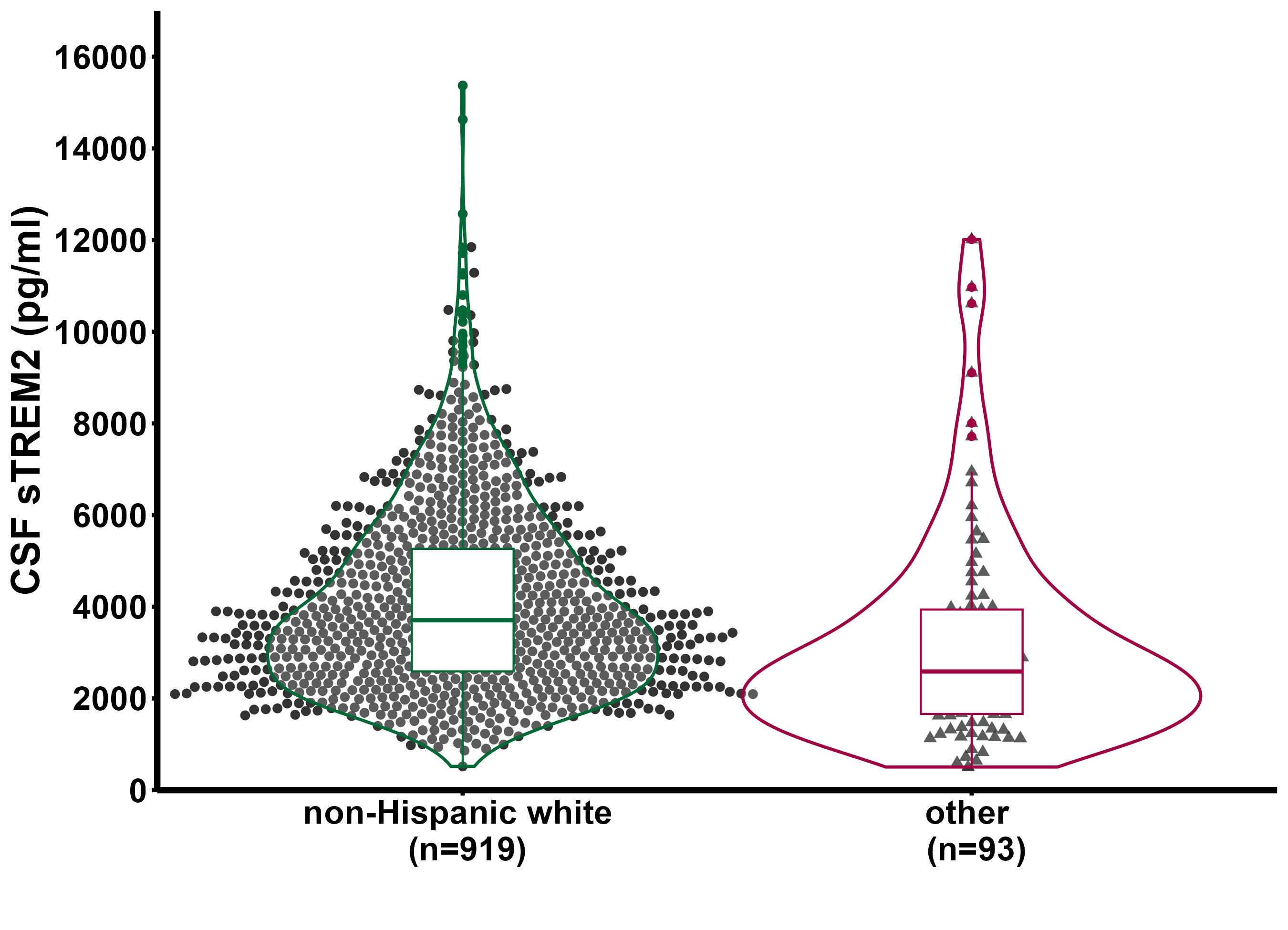


**Figure 2A. Figure 2B.**


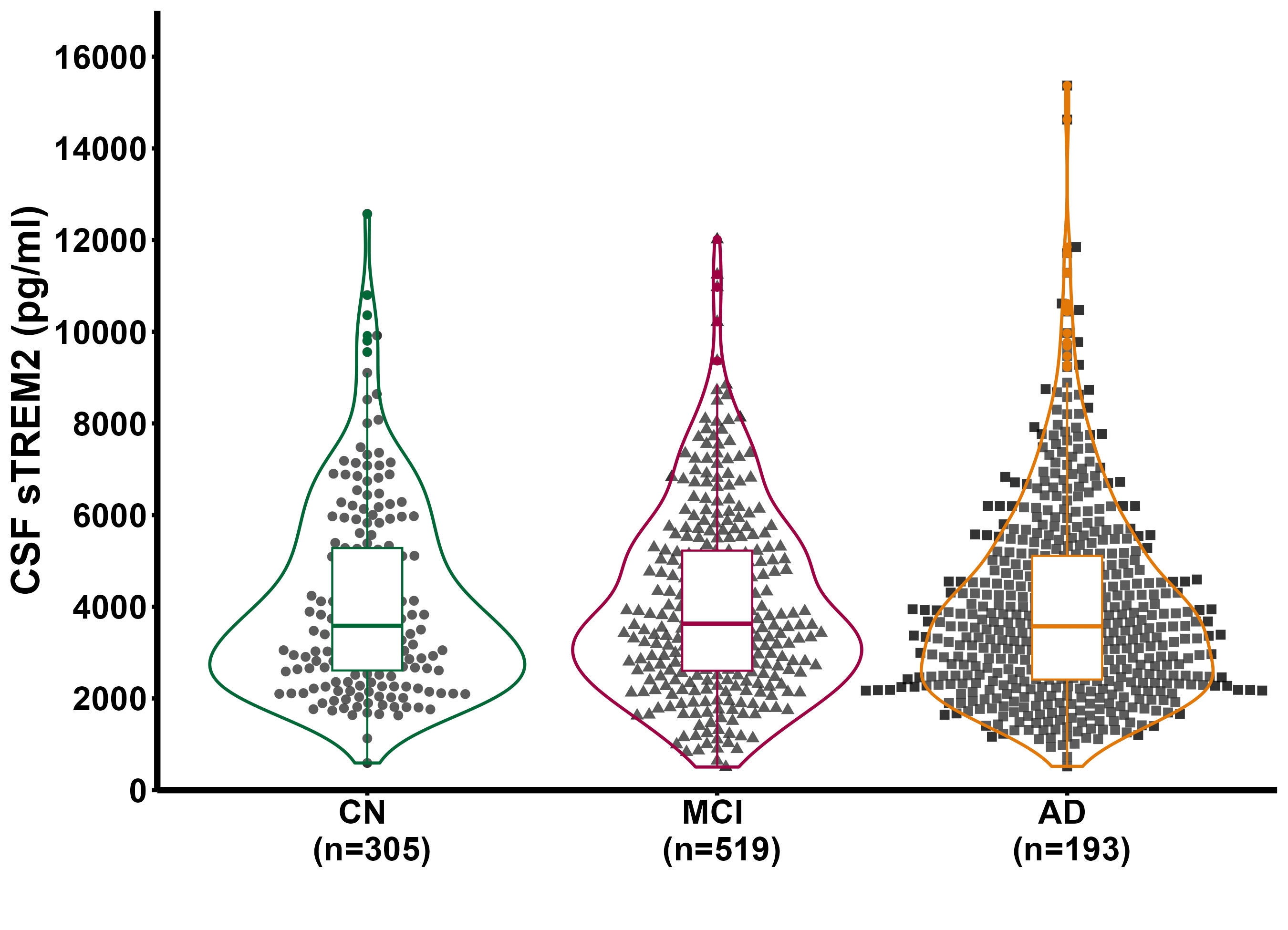

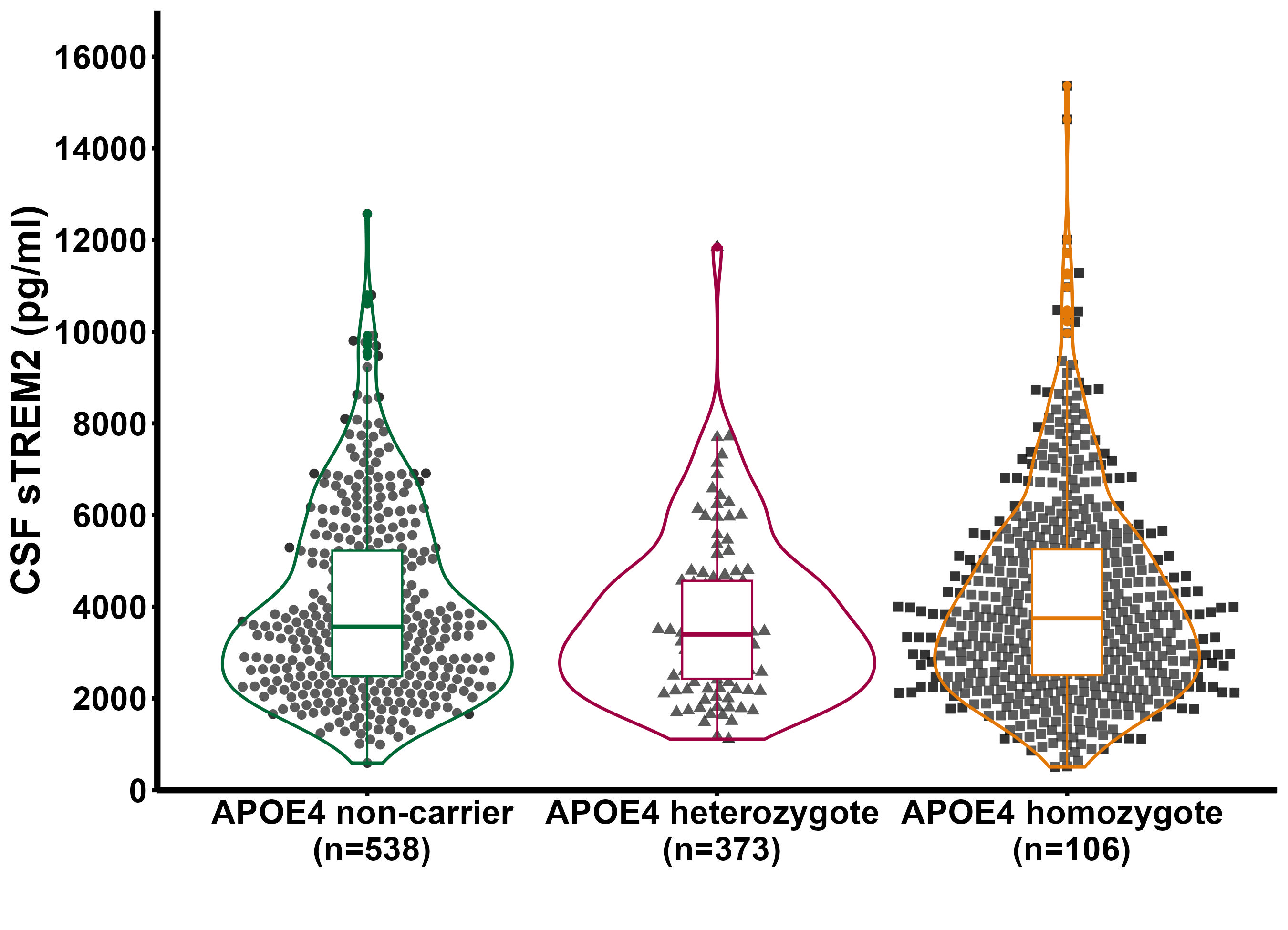


**Figure 2C. Figure 2D.**

**Figure S2A.** The distribution of CSF sTREM2 by ATN profiles at baseline. **Figure S2B.** The distribution of CSF sTREM2 by race/ethnicity at baseline. **Figure S2C.** The distribution of CSF sTREM2 by clinical cognitive status at baseline. **Figure S2D.** The distribution of CSF sTREM2 by *APOE* ε4 carrying status at baseline.

Abbreviations: CN, cognitively normal; MCI, mild cognitive impairment; CSF, cerebrospinal fluid; sTREM2, soluble TREM2; *AOPE* ε4 non-carrier, *APOE* ε2/ε2 or *APOE* ε2/ε3 or *APOE* ε3/ε2 or *APOE* ε3/ε3; *AOPE* ε4 heterozygote, *APOE* ε3/ε4 or *APOE* ε2/ε4; *AOPE* ε4 homozygote, *APOE* ε4/ε4; The ATN classification system included 3 biomarker subgroups: “A” as Aβ aggregation, “T” as tauopathy, and “N” as neurodegeneration. Aβ-positive (A+) participants were those with CSF Aβ_1-42_ levels < 976.6 pg/ml. Tau-positive (T+) participants referred to those who had a p-Tau > 21.8 pg/ml. Neurodegenerative-positive (N+) individuals were those with t-Tau > 245 pg/ml.

**Supplemental Materials: R Codes**

| library(lme4)  # "Time" is the longitudinal age at visit. "RID" is the unique subject ID.  Mydata$Time <- scale(Mydata$age, center = TRUE, scale = FALSE)  # Fit a model with random slope but no random intercept  Model.lmer1 = lmer(sTREM2~Time+(1\|RID), data= Mydata, control=lmerControl(optimizer="bobyqa"))  summary(Model.lmer1)  # Fit a model with random intercept but no random slope  Model.lmer2 = lmer(sTREM2~Time+(0+Time\|RID), data= Mydata, control=lmerControl(optimizer="bobyqa"))  summary(Model.lmer2)  # Fit a model with random intercept and random slope  Model.lmer3 = lmer(sTREM2~Time+(1+Time\|RID), data= Mydata,  control=lmerControl(optimizer="bobyqa", check.nobs.vs.nRE="ignore", calc.derivs=FALSE, check.nlev.gtr.1="ignore"))  summary(Model.lmer3)  # Fit a model with uncorrelated random intercept and slope  Model.lmer4 = lmer(sTREM2~Time+(1\|RID)+(0+Time\|RID), data= Mydata, control=lmerControl(optimizer="bobyqa"))  summary(Model.lmer4)  # The best random effects structure is determined by comparing their AIC and BIC values  anova(Model.lmer1, Model.lmer2, Model.lmer3, Model.lmer4)  # Adding covariates as fixed effect to models  Model1=lmer(sTREM2~Time+(1+Time\|RID), data= Mydata,  control=lmerControl(optimizer="bobyqa", check.nobs.vs.nRE="ignore", calc.derivs=FALSE, check.nlev.gtr.1="ignore"))  summary(Model1)  Model2=lmer(sTREM2~Time+sex+race +(1+Time\|RID), data= Mydata,  control=lmerControl(optimizer="bobyqa", check.nobs.vs.nRE="ignore", calc.derivs=FALSE, check.nlev.gtr.1="ignore"))  summary(Model2)  Model3=lmer(sTREM2~Time+sex+race+TREM2_carrying+APOE4+(1+Time\|RID), data= Mydata,  control=lmerControl(optimizer="bobyqa", check.nobs.vs.nRE="ignore", calc.derivs=FALSE, check.nlev.gtr.1="ignore"))  summary(Model13)  Model4=lmer(sTREM2~Time+sex+race+TREM2_carrying+APOE4+Educationa+Smoking + Maried+ Cognitive+(1+Time\|RID), data= Mydata, control=lmerControl(optimizer="bobyqa", check.nobs.vs.nRE="ignore", calc.derivs=FALSE, check.nlev.gtr.1="ignore"))  summary(Model4)  Model5=lmer(sTREM2~Time+ sex+race+TREM2_carrying+APOE4+Educationa+Smoking+Maried+Cognitive+Aβ_1-42_+p-Tau+t-Tau +(1+Time\|RID), data= Mydata, control=lmerControl(optimizer="bobyqa", check.nobs.vs.nRE="ignore", calc.derivs=FALSE, check.nlev.gtr.1="ignore"))  summary(Model5)  Model6=lmer(sTREM2~Time+sex+race+TREM2_carrying+APOE4+ratio_ptau_abeta+(1+Time\|RID), data= Mydata, control=lmerControl(optimizer="bobyqa", check.nobs.vs.nRE="ignore", calc.derivs=FALSE, check.nlev.gtr.1="ignore"))  summary(Model6)  # Adding interaction terms to models  Model_inter1=lmer(sTREM2~Time:Cognitive+Time+Cognitive+sex+race+TREM2_carrying+APOE4+Educationa+Smoking+Maried+Cognitive+(1+Time\|RID), data= Mydata, control=lmerControl(optimizer="bobyqa", check.nobs.vs.nRE="ignore", calc.derivs=FALSE, check.nlev.gtr.1="ignore"))  summary(Model_inter1)  Model_inter2=lmer(sTREM2~sex:Cognitive+sex+Cognitive+Time+race+TREM2_carrying+APOE4+Educationa+Smoking+Maried+Cognitive+(1+Time\|RID), data= Mydata, control=lmerControl(optimizer="bobyqa", check.nobs.vs.nRE="ignore", calc.derivs=FALSE, check.nlev.gtr.1="ignore"))  summary(Model_inter2) |
| --- |
